# Supplementary material for: Chitosan/Phosphate Rock-Derived Natural Polymeric Composite to Sequester Divalent Copper Ions from Water
Source: Nanomaterials (Basel). 2021 Aug 9;11(8):2028. doi: 10.3390/nano11082028 (PMC8400442; doi:10.3390/nano11082028)
Supplement: Supplementary file 1 [file nanomaterials-11-02028-s001.zip › nanomaterials-1316545-supplementary.pdf]

nanomaterials

MDPI

Supplementary Information

Chitosan/Phosphate Rock-Derived Natural Polymeric Composite to Sequester Divalent Copper Ions from Water

Rachid El Kaim Billah [1], Moonis Ali Khan [2,*], Saikh Mohammad Wabaidur [2], Byong-Hun Jeon [3], Amira AM [4], Hicham Majdoubi [4], Younesse Haddaji [5], Mahfoud Agunaou [1] and Abdessadik Soufiane [1]

[1] Laboratory of Coordination and Analytical Chemistry, Department of Chemistry, Faculty of Sciences, University of Chouaib Doukkali, El Jadida 24000, Morocco; rachidelkaimbillah@gmail.com (R.E.K.B.); m.agunaou@gmail.com (M.A.); abdessadiksoufiane@gmail.com (A.S.)
[2] Chemistry Department, College of Science, King Saud University, Riyadh 11451, Saudi Arabia; swabaidur@ksu.edu.sa
[3] Department of Earth Resources and Environmental Engineering, Hanyang University, Seoul 04763, Korea; bhjeon@hanyang.ac.kr
[4] Laboratory of Analytical Chemistry and Physico-Chemistry of Materials, Department of Chemistry, Faculty of Sciences Ben M'Sik, University of Hassan II-Casablanca, Casablanca 21100, Morocco; Amamira6@gmail.com (A.A.); hichammajdoubi.hm@gmail.com (H.M.)
[5] Laboratory of Engineering and Materials, Department of Chemistry, Faculty of Sciences Ben M'Sik, University of Hassan II-Casablanca, Casablanca 21100, Morocco; ys.haddaji@gmail.com
* Correspondence: mokhan@ksu.edu.sa

Text S1. Isotherm models

Langmuir isotherm model assumes formation of monomolecular layer over adsorbent surface without interaction between the adsorbed molecules. Langmuir isotherm model [1] in linearized form is expressed as:

$$\frac{1}{q_e} = \frac{1}{q_m} + \frac{1}{q_m K_L} \times \frac{1}{C_e} \quad \text{(S1)}$$

where q_m (mg/g) and K_L (L/mg) are the constants for maximum monolayer adsorption capacity and a constant related to the heat of adsorption, respectively.

Freundlich isotherm [2] in linearized form is expressed as:

$$\ln q_e = \ln K_F + \frac{1}{n} \ln C_e \quad \text{(S2)}$$

where K_F ((mg/g) (L/mg)$^{(1/n)}$) and n are the Freundlich constants related to bonding energy and deviation in adsorption from linearity, respectively.

Text S2. Adsorption kinetic models

The pseudo-first-order [3], and pseudo-second-order [4] kinetic models in linearized forms are expressed as:

$$\log(q_{e1} - q_t) = \log q_e - \frac{k_1}{2.303} \times t \quad \text{(S3)}$$

$$\frac{t}{q_t} = \frac{1}{k_2 q_{e2}^2} + \frac{1}{q_e} \times t \quad \text{(S4)}$$

where q_{e1}, q_{e2} and q_t are the adsorption capacities for pseudo-first order model, pseudo-second order model at equilibriums, and at time t, respectively, k_1 (1/min) and k_2 (g/mg-min) are the pseudo-first-order and pseudo-second-order rate constants, respectively.

Text S3. Thermodynamic parameters

Thermodynamic parameters viz. standard Gibb's free energy change ($\Delta G°$), entropy change ($\Delta S°$), and enthalpy change ($\Delta H°$) were calculated as:

$$\Delta G° = -RT \ln K_c \quad (S5)$$

$$\ln K_c = \frac{\Delta S°}{R} - \frac{\Delta H°}{R} \times \frac{1}{T} \quad (S6)$$

where R is the universal gas constant (8.314 J/mol-K), T is the absolute temperature (K), and K_c is a separation factor. $\Delta S°$ and $\Delta H°$ were calculated from the intercept and slope of the plot of ln K_c versus $1/T$.

References

1. Langmuir, I. The adsorption of gases on plane surface of glass, mica andplatinum. *J. Am. Chem. Soc.* **1916**, *40*, 1361–1403.
2. Freundlich, H.M.F. Over the adsorption in solution. *J. Phys. Chem.* **1906**, *57*, 385–470.
3. Lagergren, S.K. About the theory of so-called adsorption of soluble substances. *Sven. Vetenskapsakad. Handl. 1989*, **24**, 1–39.
4. Ho, Y.S.; McKay, G. The kinetics of sorption of divalent metal ions ontosphagnum moss peat. *Water Res.* **2000**, *34*, 735–742.
